# Supplementary material for: Optimising supervised machine learning algorithms predicting cigarette cravings and lapses for a smoking cessation just-in-time adaptive intervention (JITAI)
Source: PLoS One. 2026 May 14;21(5):e0349028. doi: 10.1371/journal.pone.0349028 (PMC13175349; doi:10.1371/journal.pone.0349028)
Supplement: S3 Appendix — (DOCX) [file pone.0349028.s003.docx]

## Description of observations among the participants with at least one lapse

| Prompts per day | EMAs | | | | Lapses | | | | High cravings | | | |
| --- | --- | --- | --- | --- | --- | --- | --- | --- | --- | --- | --- | --- |
|  | Number | | | Proportion event contingent | Overall proportion | Across individuals | | | Overall proportion | Across individuals | | |
|  | Total | Event contingent | Signal contingent |  |  | Median proportion | 25th percentile proportion | 75th percentile proportion |  | Median proportion | 25th percentile proportion | 75th percentile proportion |
| 16 | 4,044 | 44 | 4,000 | 1.1% | 10.2% | 6.2% | 1.2% | 13.4% | 34.6% | 29.6% | 13.1% | 44.1% |
| 6 | 1,483 | 43 | 1,440 | 2.9% | 19.0% | 12.3% | 3.3% | 28.6% | 35.0% | 28.8% | 13.8% | 42.7% |
| 5 | 1,243 | 43 | 1,200 | 3.5% | 21.6% | 13.6% | 2.0% | 32.6% | 35.4% | 31.1% | 16.6% | 45.6% |
| 4 | 1,003 | 43 | 960 | 4.3% | 24.5% | 15.5% | 4.3% | 40.2% | 37.8% | 33.0% | 21.1% | 47.5% |
| 3 | 763 | 43 | 720 | 5.6% | 28.7% | 18.6% | 5.7% | 43.4% | 37.5% | 32.4% | 16.5% | 45.5% |

## Description of observations among the participants with at least one lapse beyond day 1

| Prompts per day | EMAs | | | | Lapses | | | | High cravings | | | |
| --- | --- | --- | --- | --- | --- | --- | --- | --- | --- | --- | --- | --- |
|  | Number | | | Proportion event contingent | Overall proportion | Across individuals | | | Overall proportion | Across individuals | | |
|  | Total | Event contingent | Signal contingent |  |  | Median proportion | 25th percentile proportion | 75th percentile proportion |  | Median proportion | 25th percentile proportion | 75th percentile proportion |
| 16 | 3,241 | 41 | 3,200 | 1.3% | 12.6% | 7.4% | 4.2% | 17.5% | 34.6% | 29.7% | 12.6% | 42.4% |
| 6 | 1,241 | 41 | 1,200 | 3.3% | 22.3% | 14.8% | 7.8% | 31.9% | 36.3% | 30.5% | 13.8% | 42.7% |
| 5 | 1,041 | 41 | 1,000 | 3.9% | 25.5% | 17.6% | 8.8% | 38.3% | 36.4% | 32.7% | 16.6% | 45.6% |
| 4 | 841 | 41 | 800 | 4.9% | 28.8% | 20.7% | 9.7% | 43.5% | 39.4% | 35.3% | 22.8% | 47.5% |
| 3 | 611 | 41 | 570 | 6.7% | 35.0% | 29.0% | 14.3% | 52.4% | 36.0% | 37.5% | 17.4% | 43.5% |

## Description of observations among the participants with at least one lapse beyond day 2

| Prompts per day | EMAs | | | | Lapses | | | | High cravings | | | |
| --- | --- | --- | --- | --- | --- | --- | --- | --- | --- | --- | --- | --- |
|  | Number | | | Proportion event contingent | Overall proportion | Across individuals | | | Overall proportion | Across individuals | | |
|  | Total | Event contingent | Signal contingent |  |  | Median proportion | 25th percentile proportion | 75th percentile proportion |  | Median proportion | 25th percentile proportion | 75th percentile proportion |
| 16 | 2,759 | 39 | 2,720 | 1.4% | 14.6% | 10.2% | 6.2% | 18.4% | 29.6% | 29.6% | 13.1% | 36.2% |
| 6 | 1,059 | 39 | 1,020 | 3.7% | 25.8% | 23.8% | 11.8% | 37.7% | 31.4% | 29.7% | 14.5% | 38.7% |
| 5 | 889 | 39 | 850 | 4.4% | 29.5% | 24.5% | 13.5% | 45.1% | 32.2% | 31.4% | 17.0% | 40.0% |
| 4 | 719 | 39 | 680 | 5.4% | 33.1% | 30.2% | 14.3% | 51.2% | 35.0% | 34.1% | 23.3% | 42.9% |
| 3 | 549 | 39 | 510 | 7.1% | 38.4% | 33.3% | 16.1% | 54.8% | 34.2% | 37.5% | 18.8% | 41.9% |

## Description of observations among the participants with at least one lapse beyond day 3

| Prompts per day | EMAs | | | | Lapses | | | | High cravings | | | |
| --- | --- | --- | --- | --- | --- | --- | --- | --- | --- | --- | --- | --- |
|  | Number | | | Proportion event contingent | Overall proportion | Across individuals | | | Overall proportion | Across individuals | | |
|  | Total | Event contingent | Signal contingent |  |  | Median proportion | 25th percentile proportion | 75th percentile proportion |  | Median proportion | 25th percentile proportion | 75th percentile proportion |
| 16 | 2,759 | 39 | 2,720 | 1.4% | 14.6% | 10.2% | 6.2% | 18.4% | 29.6% | 29.6% | 13.1% | 36.2% |
| 6 | 1,059 | 39 | 1,020 | 3.7% | 25.8% | 23.8% | 11.8% | 37.7% | 31.4% | 29.7% | 14.5% | 38.7% |
| 5 | 889 | 39 | 850 | 4.4% | 29.5% | 24.5% | 13.5% | 45.1% | 32.2% | 31.4% | 17.0% | 40.0% |
| 4 | 719 | 39 | 680 | 5.4% | 33.1% | 30.2% | 14.3% | 51.2% | 35.0% | 34.1% | 23.3% | 42.9% |
| 3 | 549 | 39 | 510 | 7.1% | 38.4% | 33.3% | 16.1% | 54.8% | 34.2% | 37.5% | 18.8% | 41.9% |

## Description of observations among the participants with at least one instance of high cravings beyond day 1

| Prompts per day | EMAs | | | | Lapses | | | | High cravings | | | |
| --- | --- | --- | --- | --- | --- | --- | --- | --- | --- | --- | --- | --- |
|  | Number | | | Proportion event contingent | Overall proportion | Across individuals | | | Overall proportion | Across individuals | | |
|  | Total | Event contingent | Signal contingent |  |  | Median proportion | 25th percentile proportion | 75th percentile proportion |  | Median proportion | 25th percentile proportion | 75th percentile proportion |
| 16 | 5,964 | 44 | 5,920 | 0.7% | 6.9% | 1.2% | 0.0% | 7.5% | 35.8% | 29.6% | 11.1% | 52.8% |
| 6 | 2,143 | 43 | 2,100 | 2.0% | 13.1% | 3.3% | 0.0% | 19.3% | 39.0% | 31.3% | 15.8% | 54.9% |
| 5 | 1,742 | 42 | 1,700 | 2.4% | 15.3% | 2.0% | 0.0% | 22.8% | 40.1% | 34.3% | 18.0% | 55.9% |
| 4 | 1,484 | 44 | 1,440 | 3.0% | 16.6% | 3.7% | 0.0% | 24.0% | 39.5% | 33.0% | 21.1% | 61.6% |
| 3 | 1,124 | 44 | 1,080 | 3.9% | 19.5% | 4.9% | 0.0% | 30.1% | 39.1% | 36.7% | 16.5% | 50.8% |

## Description of observations among the participants with at least one instance of high cravings beyond day 2

| Prompts per day | EMAs | | | | Lapses | | | | High cravings | | | |
| --- | --- | --- | --- | --- | --- | --- | --- | --- | --- | --- | --- | --- |
|  | Number | | | Proportion event contingent | Overall proportion | Across individuals | | | Overall proportion | Across individuals | | |
|  | Total | Event contingent | Signal contingent |  |  | Median proportion | 25th percentile proportion | 75th percentile proportion |  | Median proportion | 25th percentile proportion | 75th percentile proportion |
| 16 | 5,804 | 44 | 5,760 | 0.8% | 7.1% | 1.6% | 0.0% | 8.1% | 36.8% | 29.7% | 12.6% | 53.6% |
| 6 | 2,143 | 43 | 2,100 | 2.0% | 13.1% | 3.3% | 0.0% | 19.3% | 39.0% | 31.3% | 15.8% | 54.9% |
| 5 | 1,742 | 42 | 1,700 | 2.4% | 15.3% | 2.0% | 0.0% | 22.8% | 40.1% | 34.3% | 18.0% | 55.9% |
| 4 | 1,484 | 44 | 1,440 | 3.0% | 16.6% | 3.7% | 0.0% | 24.0% | 39.5% | 33.0% | 21.1% | 61.6% |
| 3 | 1,124 | 44 | 1,080 | 3.9% | 19.5% | 4.9% | 0.0% | 30.1% | 39.1% | 36.7% | 16.5% | 50.8% |

## Description of observations among the participants with at least one instance of high cravings beyond day 3

| Prompts per day | EMAs | | | | Lapses | | | | High cravings | | | |
| --- | --- | --- | --- | --- | --- | --- | --- | --- | --- | --- | --- | --- |
|  | Number | | | Proportion event contingent | Overall proportion | Across individuals | | | Overall proportion | Across individuals | | |
|  | Total | Event contingent | Signal contingent |  |  | Median proportion | 25th percentile proportion | 75th percentile proportion |  | Median proportion | 25th percentile proportion | 75th percentile proportion |
| 16 | 5,804 | 44 | 5,760 | 0.8% | 7.1% | 1.6% | 0.0% | 8.1% | 36.8% | 29.7% | 12.6% | 53.6% |
| 6 | 2,143 | 43 | 2,100 | 2.0% | 13.1% | 3.3% | 0.0% | 19.3% | 39.0% | 31.3% | 15.8% | 54.9% |
| 5 | 1,742 | 42 | 1,700 | 2.4% | 15.3% | 2.0% | 0.0% | 22.8% | 40.1% | 34.3% | 18.0% | 55.9% |
| 4 | 1,484 | 44 | 1,440 | 3.0% | 16.6% | 3.7% | 0.0% | 24.0% | 39.5% | 33.0% | 21.1% | 61.6% |
| 3 | 1,124 | 44 | 1,080 | 3.9% | 19.5% | 4.9% | 0.0% | 30.1% | 39.1% | 36.7% | 16.5% | 50.8% |
