# Supplementary material for: Optimising supervised machine learning algorithms predicting cigarette cravings and lapses for a smoking cessation just-in-time adaptive intervention (JITAI)
Source: PLoS One. 2026 May 14;21(5):e0349028. doi: 10.1371/journal.pone.0349028 (PMC13175349; doi:10.1371/journal.pone.0349028)

Time-varying variables distributions in full datasets (16 EMA prompts/day)

# Categorical predictors

## Alcohol use
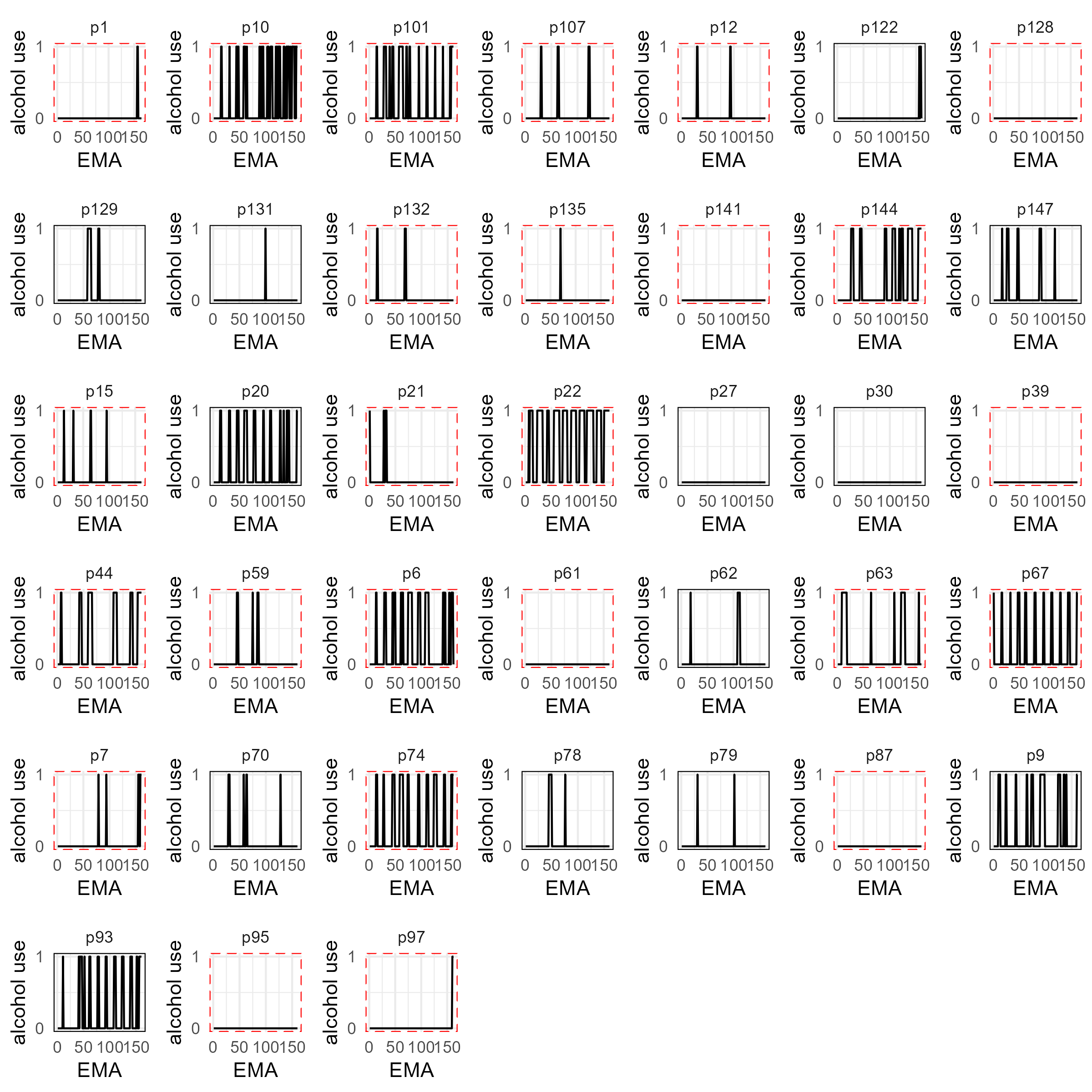


## Caffeine use


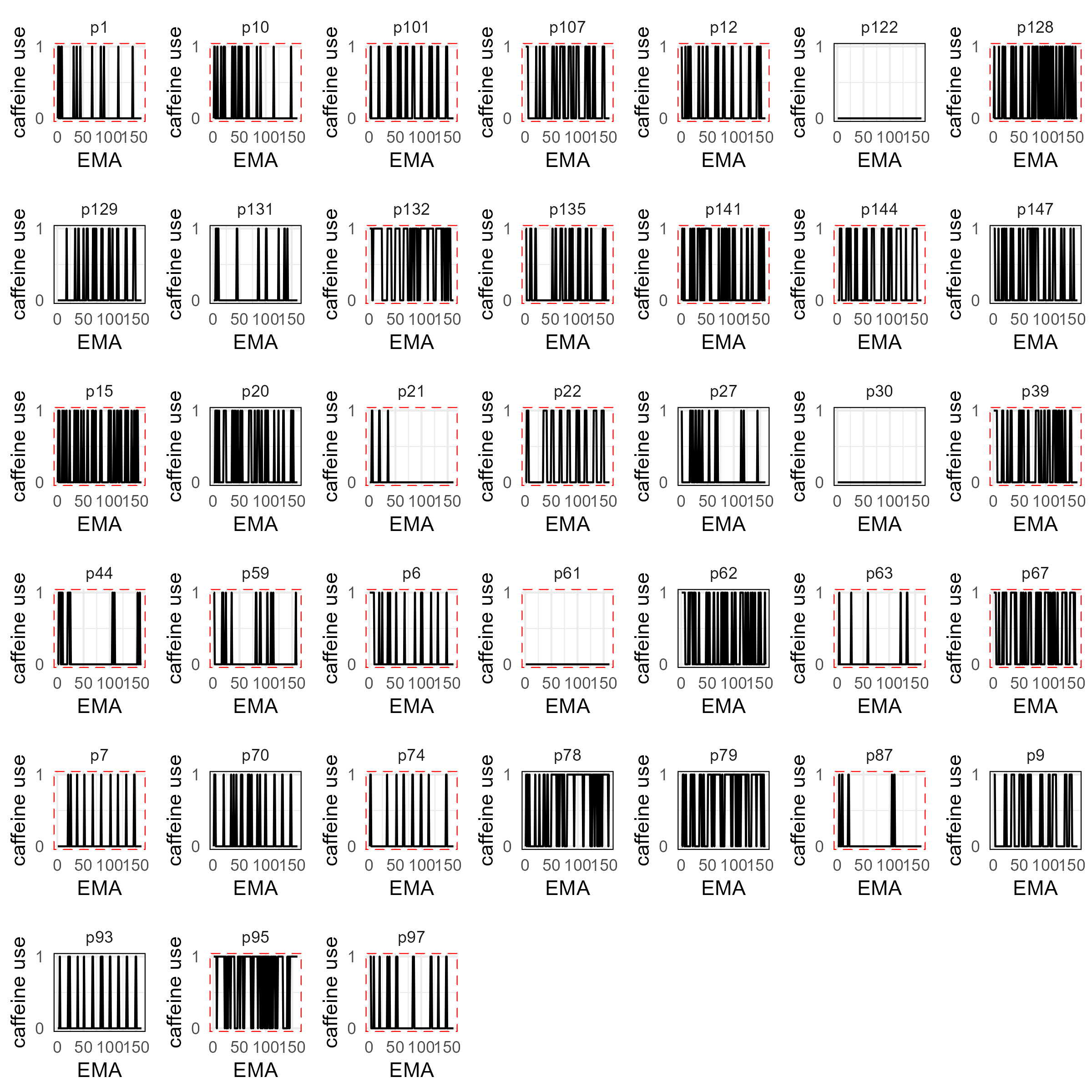


## Cigarette availability


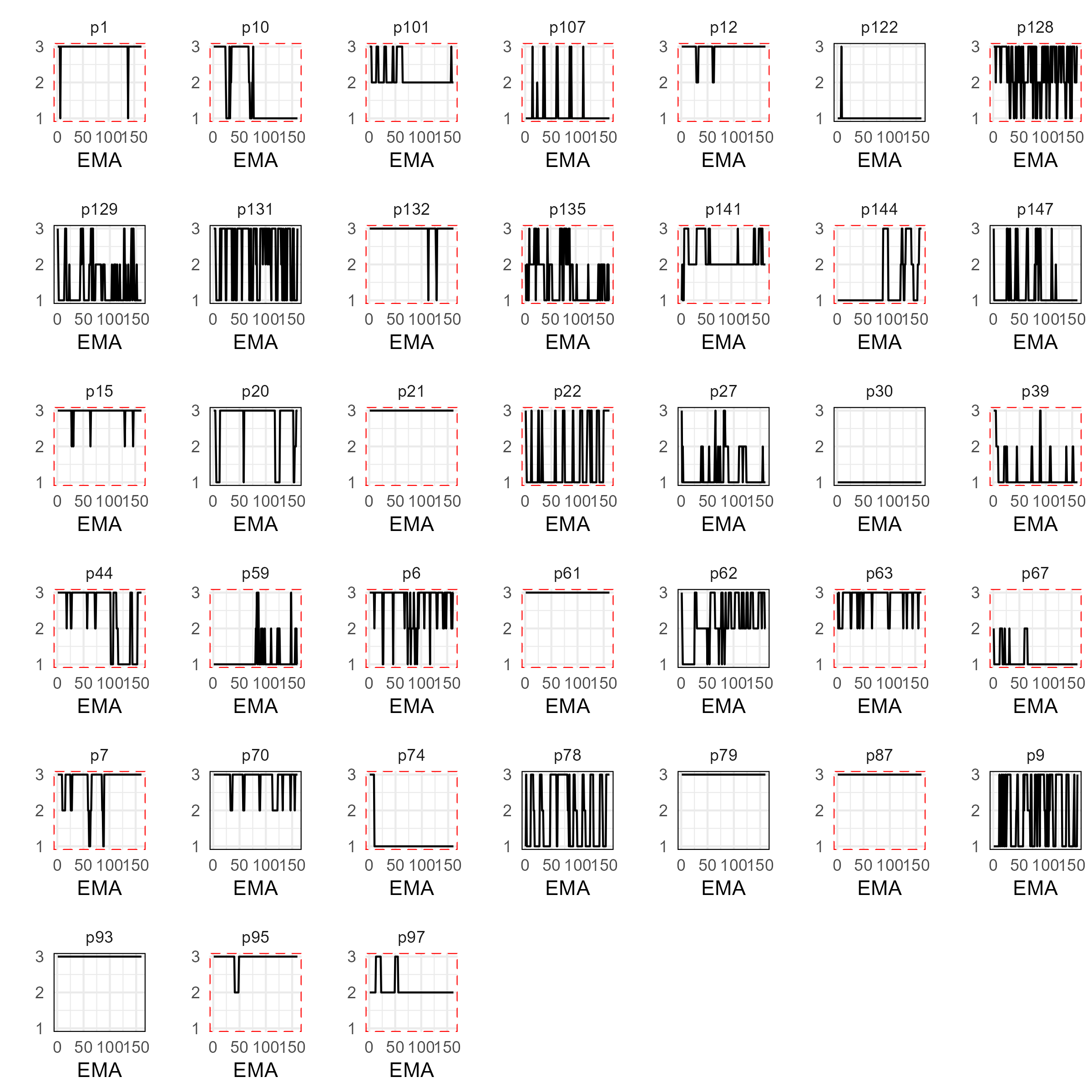


## Nicotine use


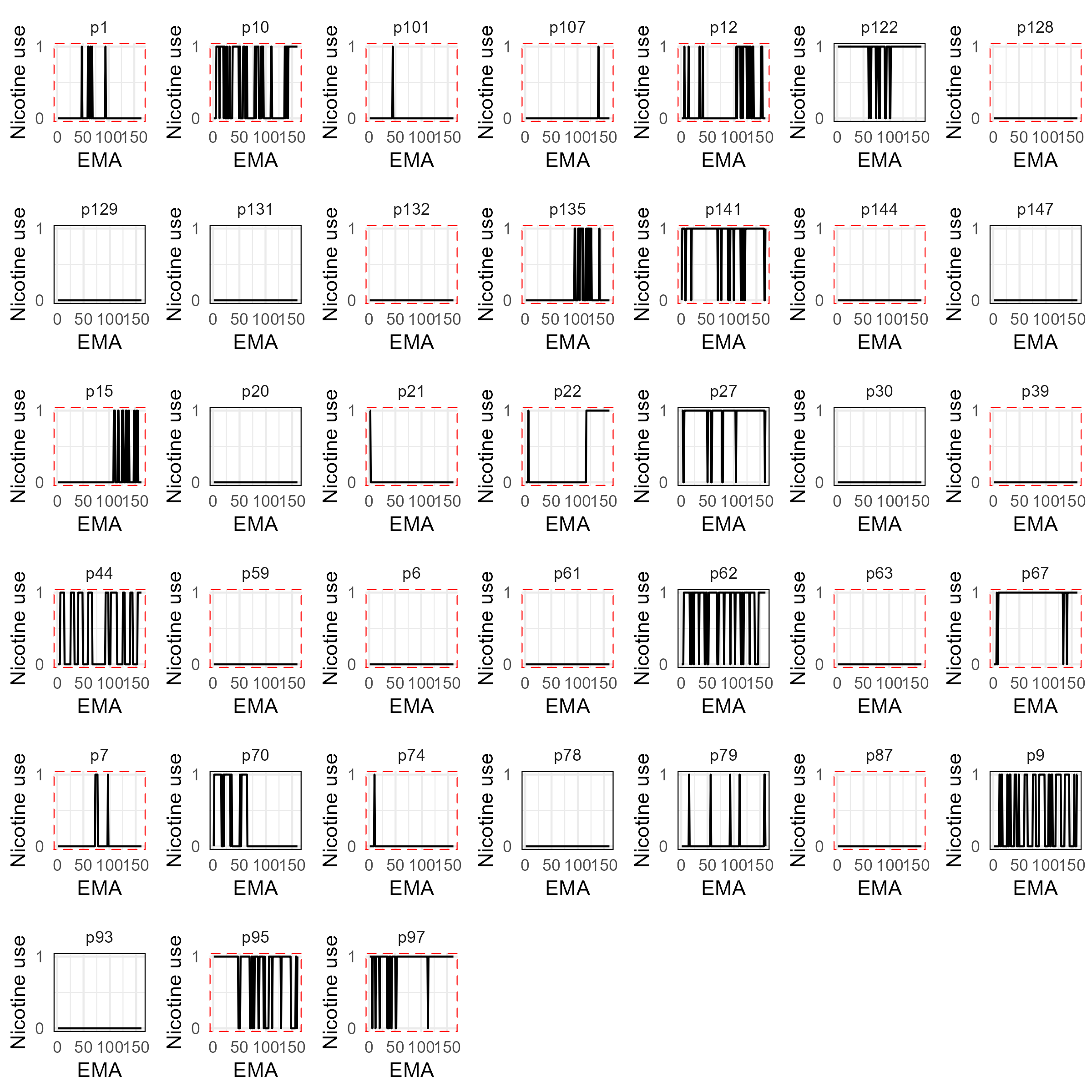


## High cravings


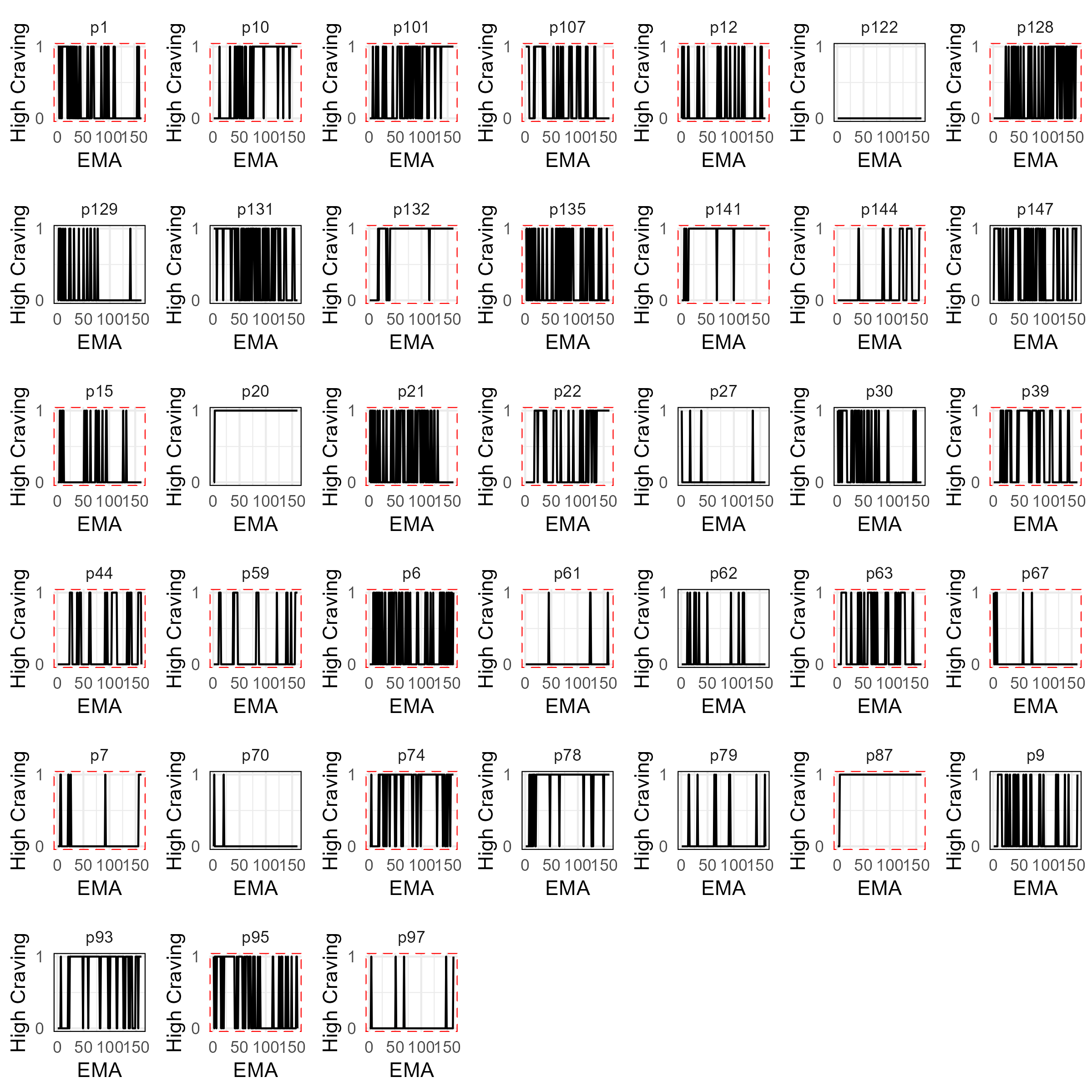


## Lapses


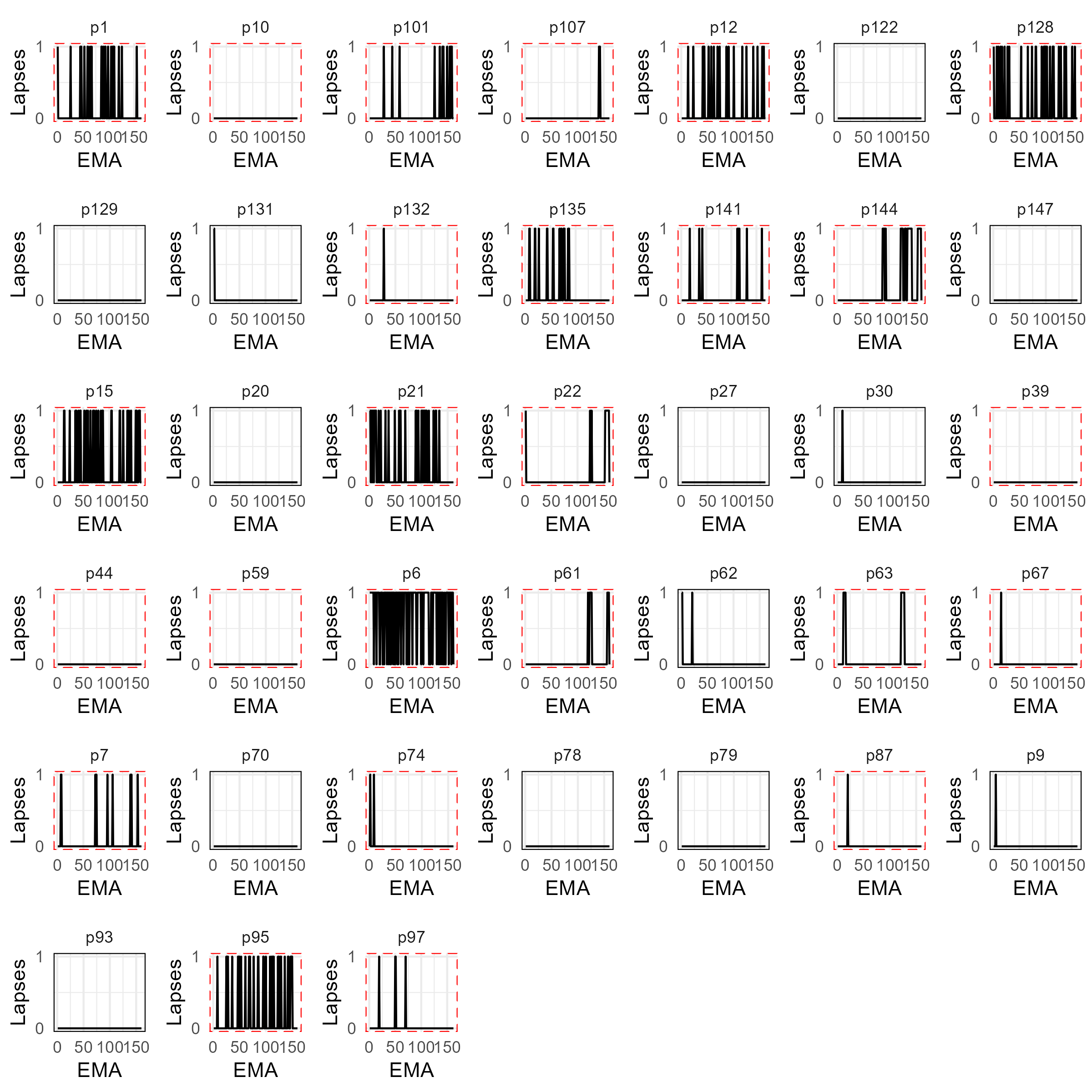


# Continuous predictors

## Anxiety


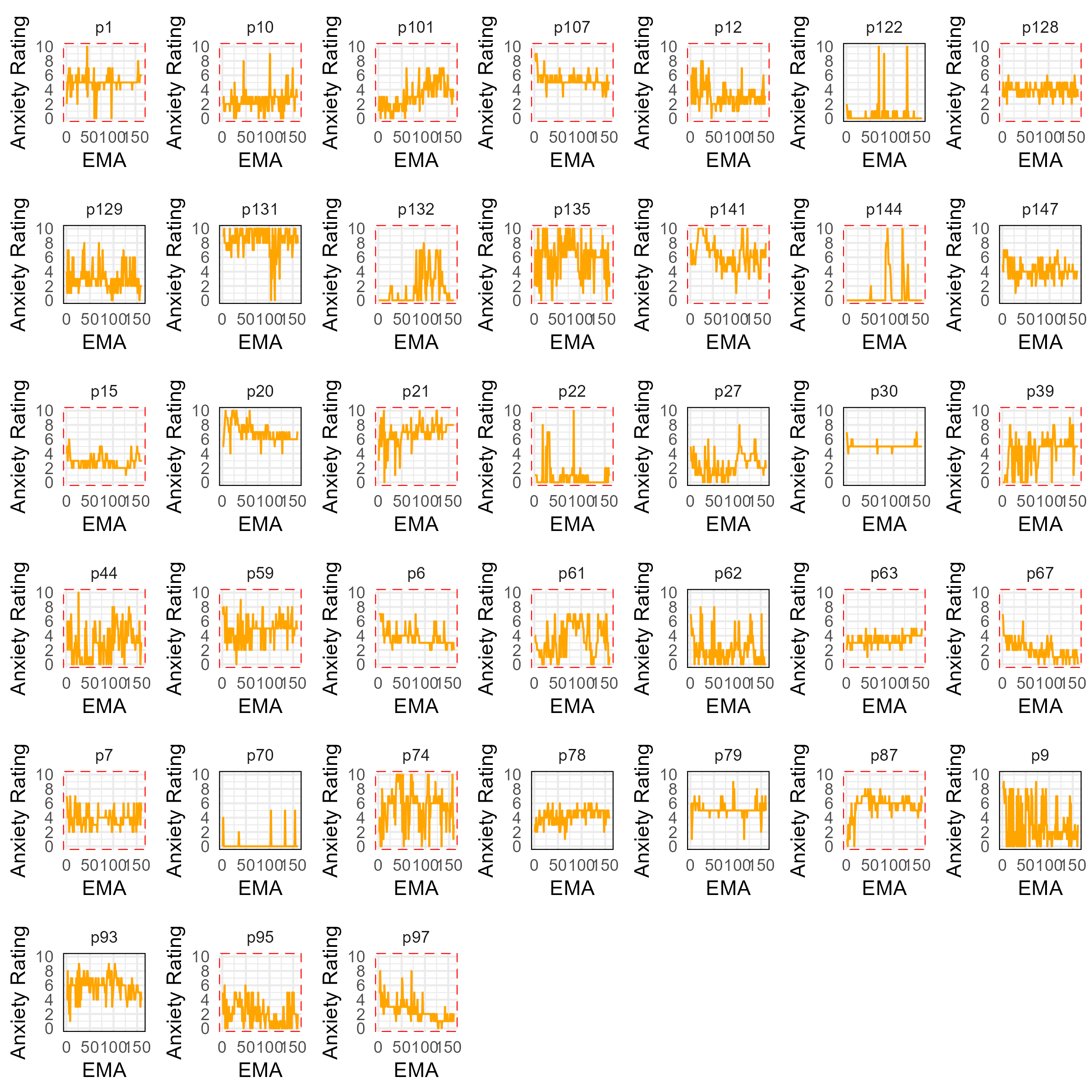


## Boredom


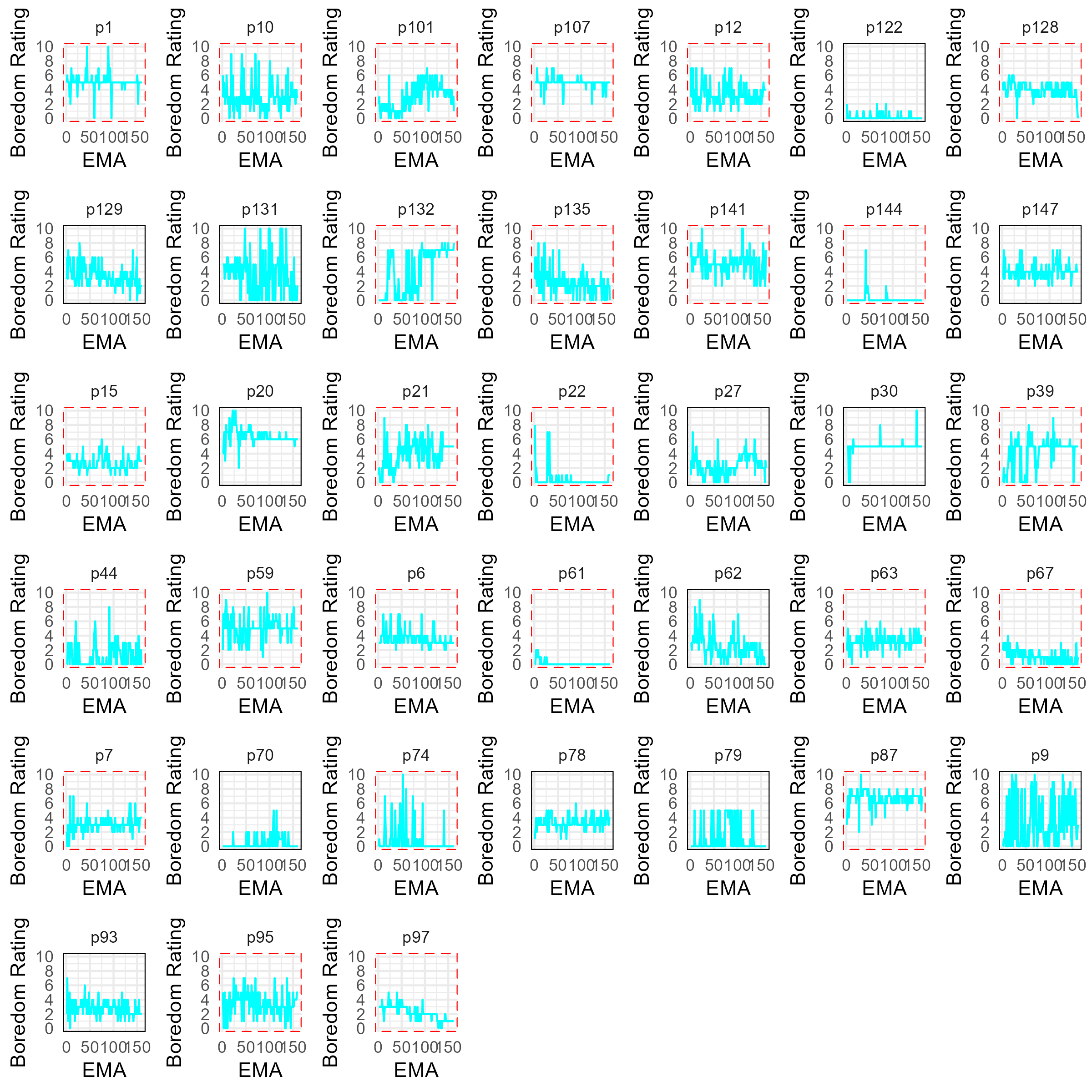


## Calmness


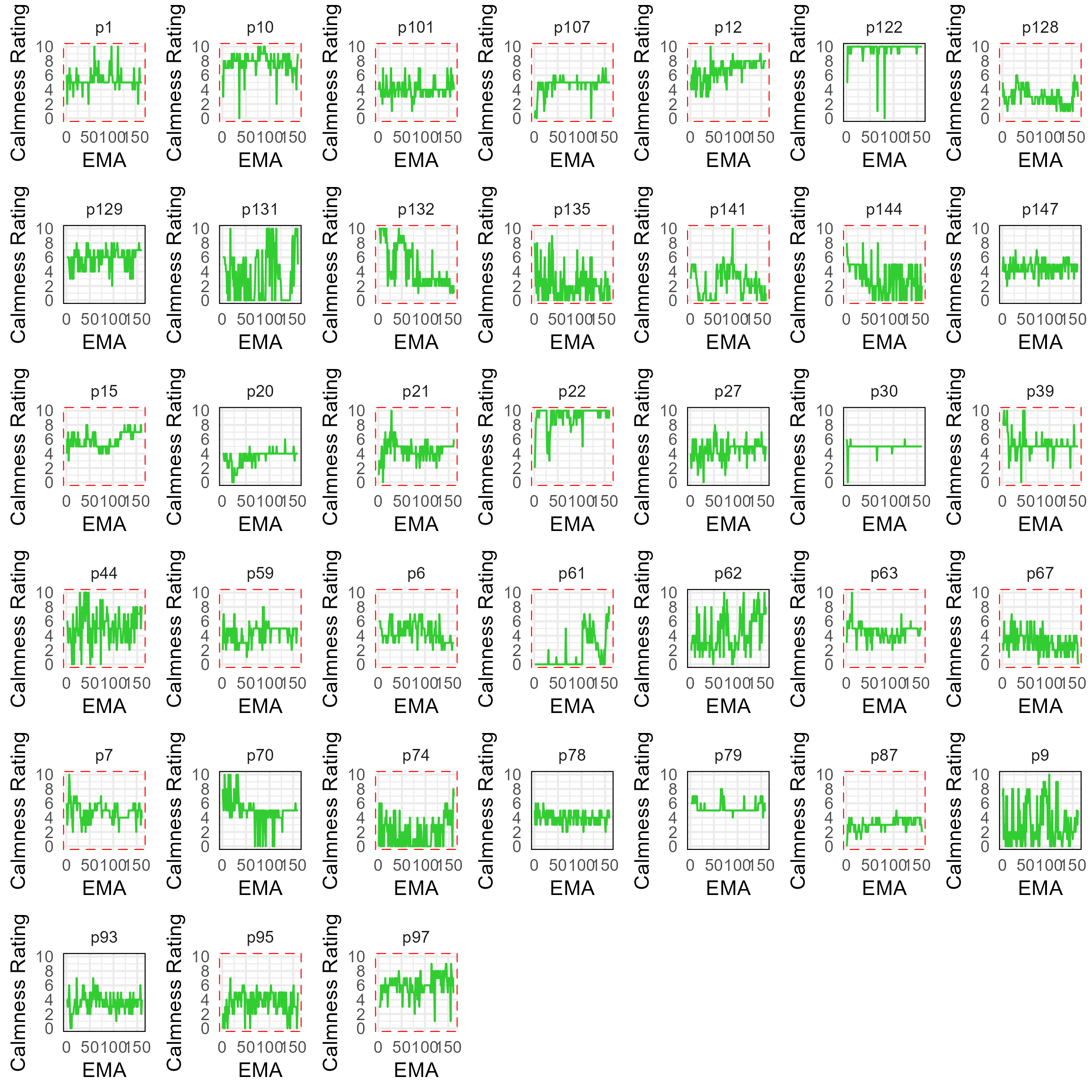


## Self-efficacy


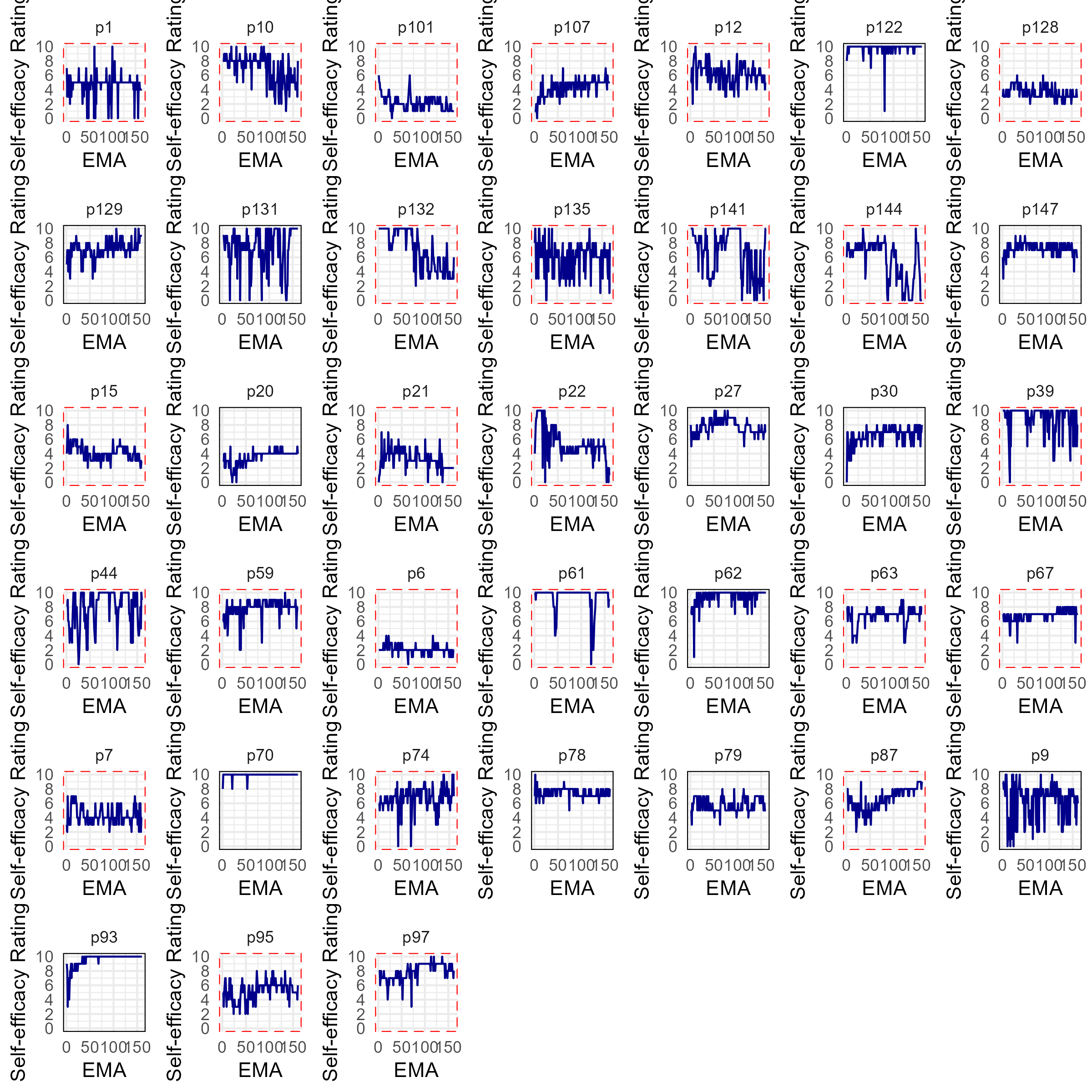


## Contended


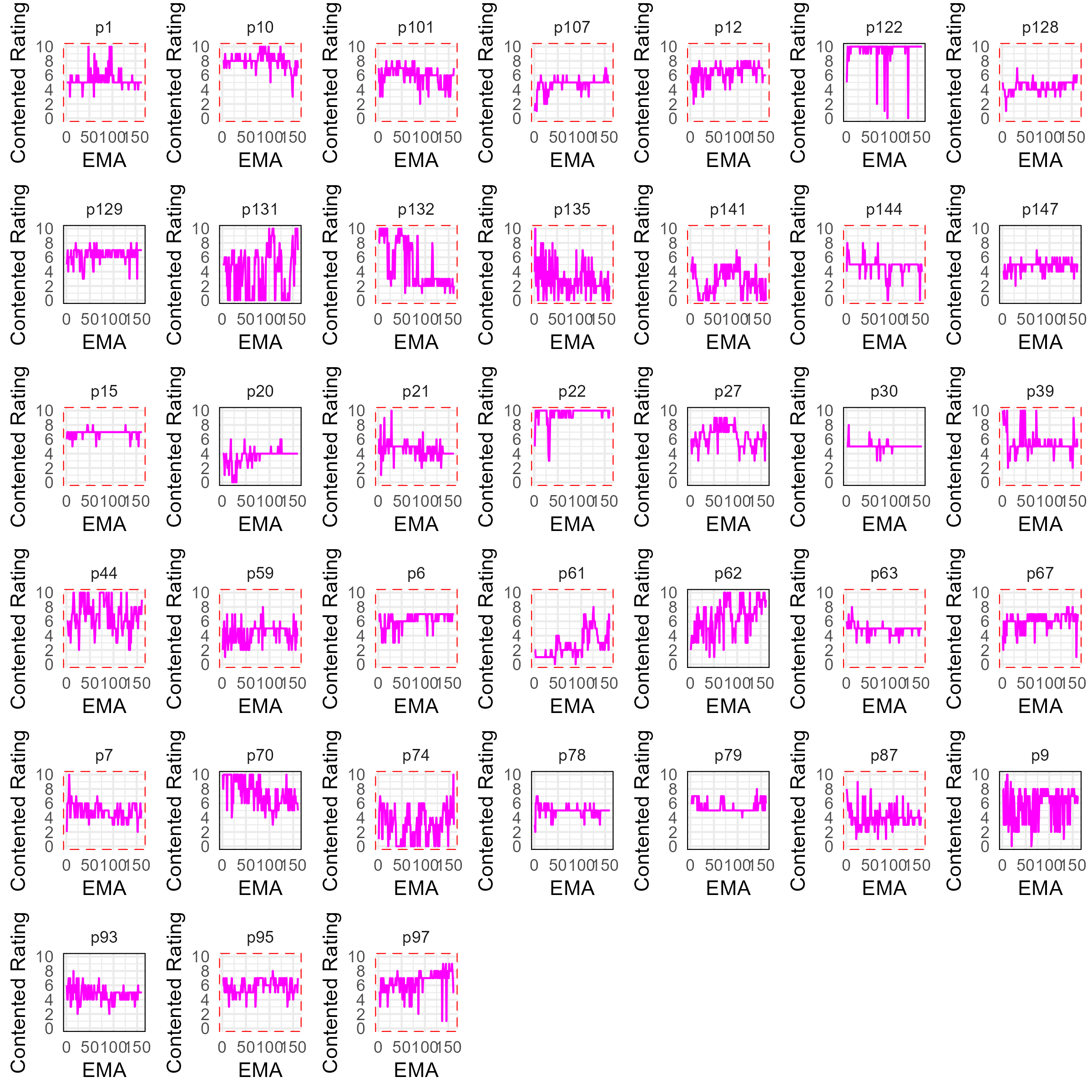


## Cravings


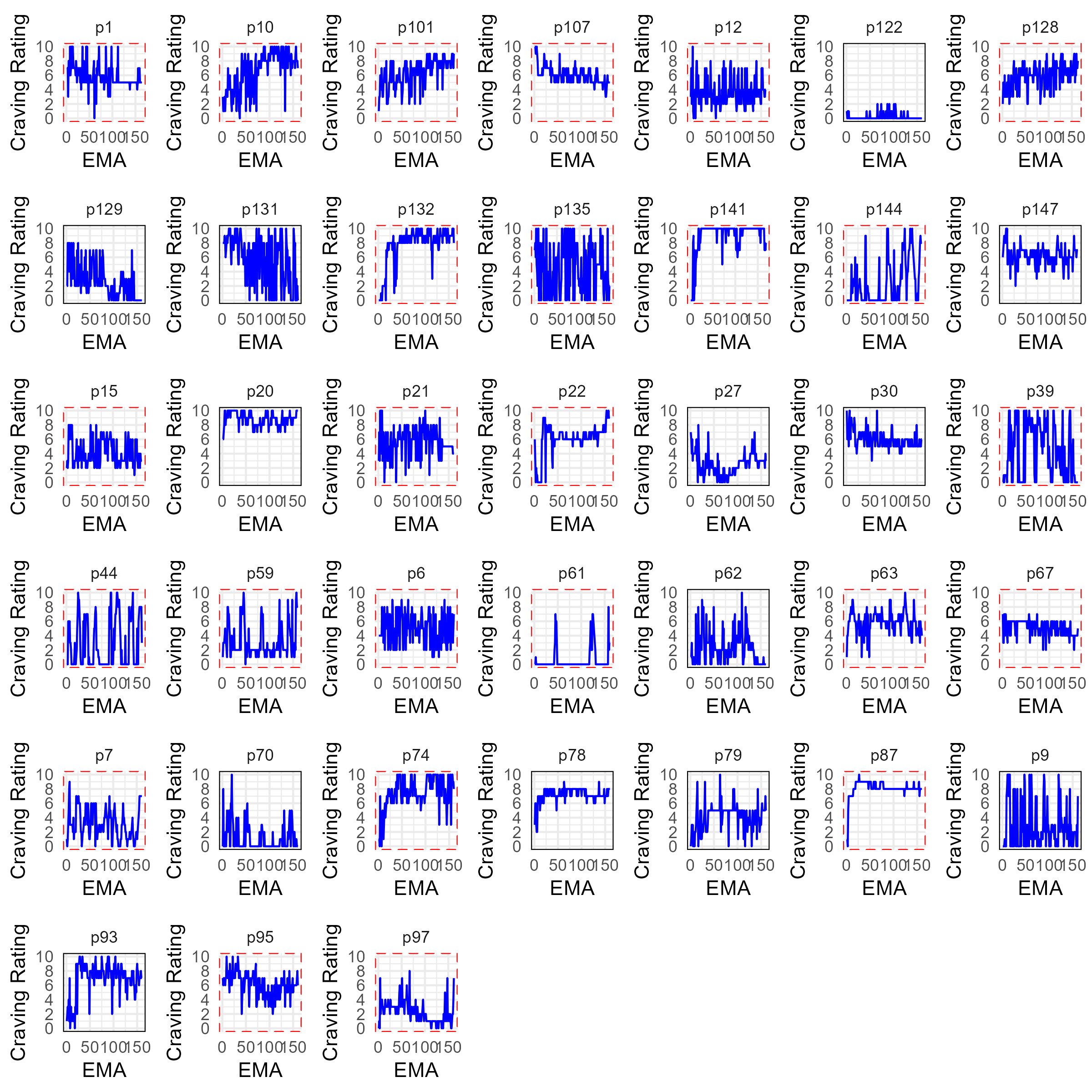


## Enthusiasm


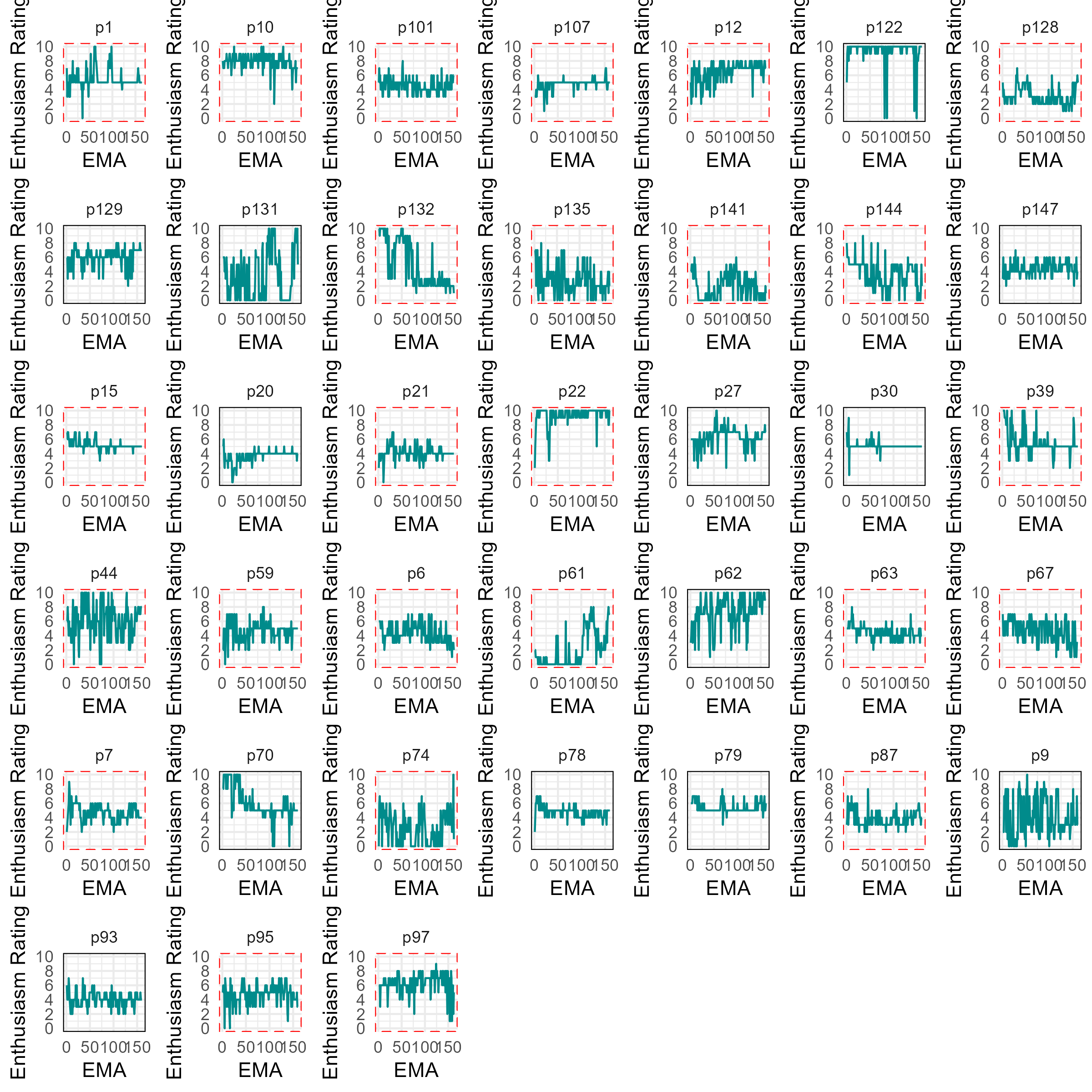


## Excitement


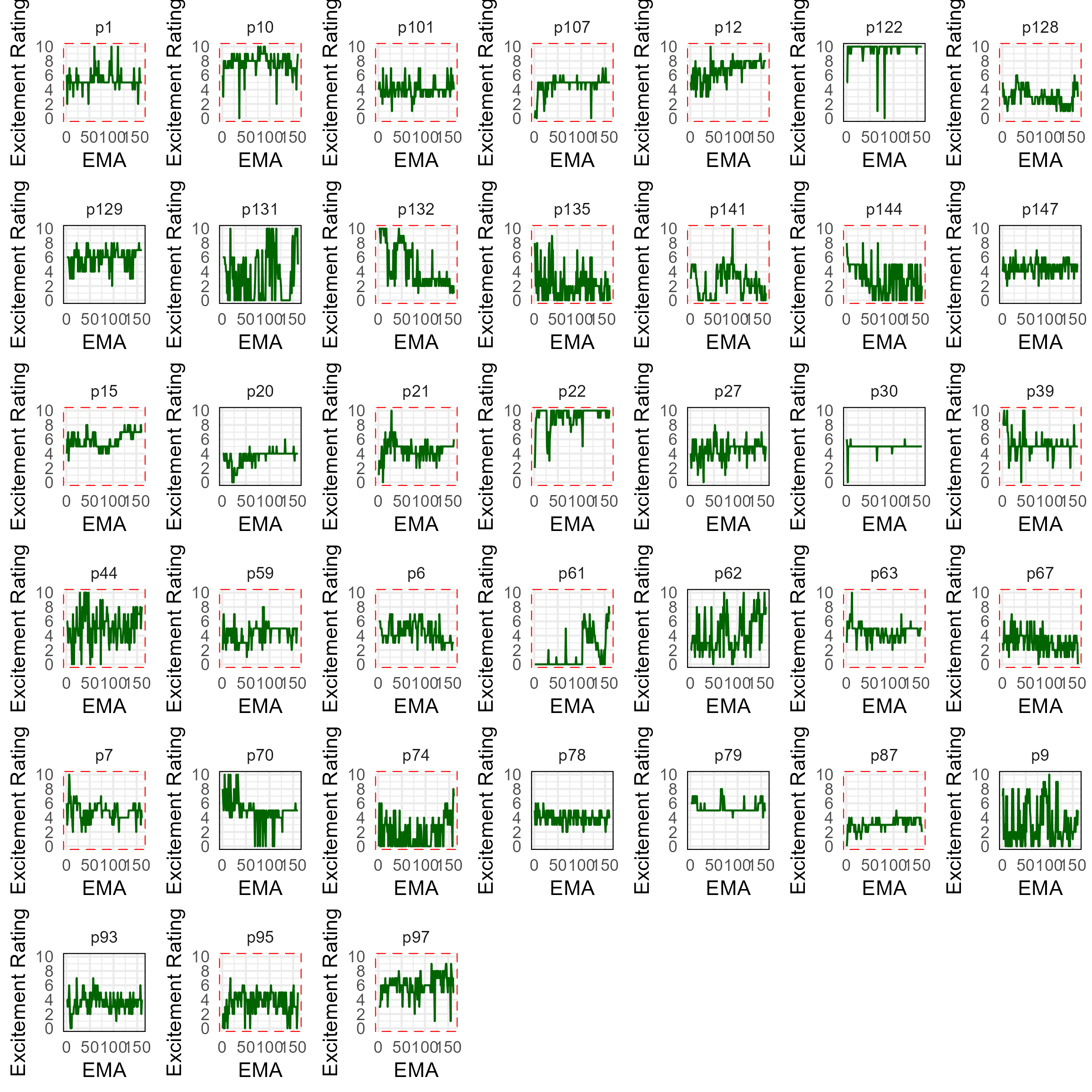


## Happiness


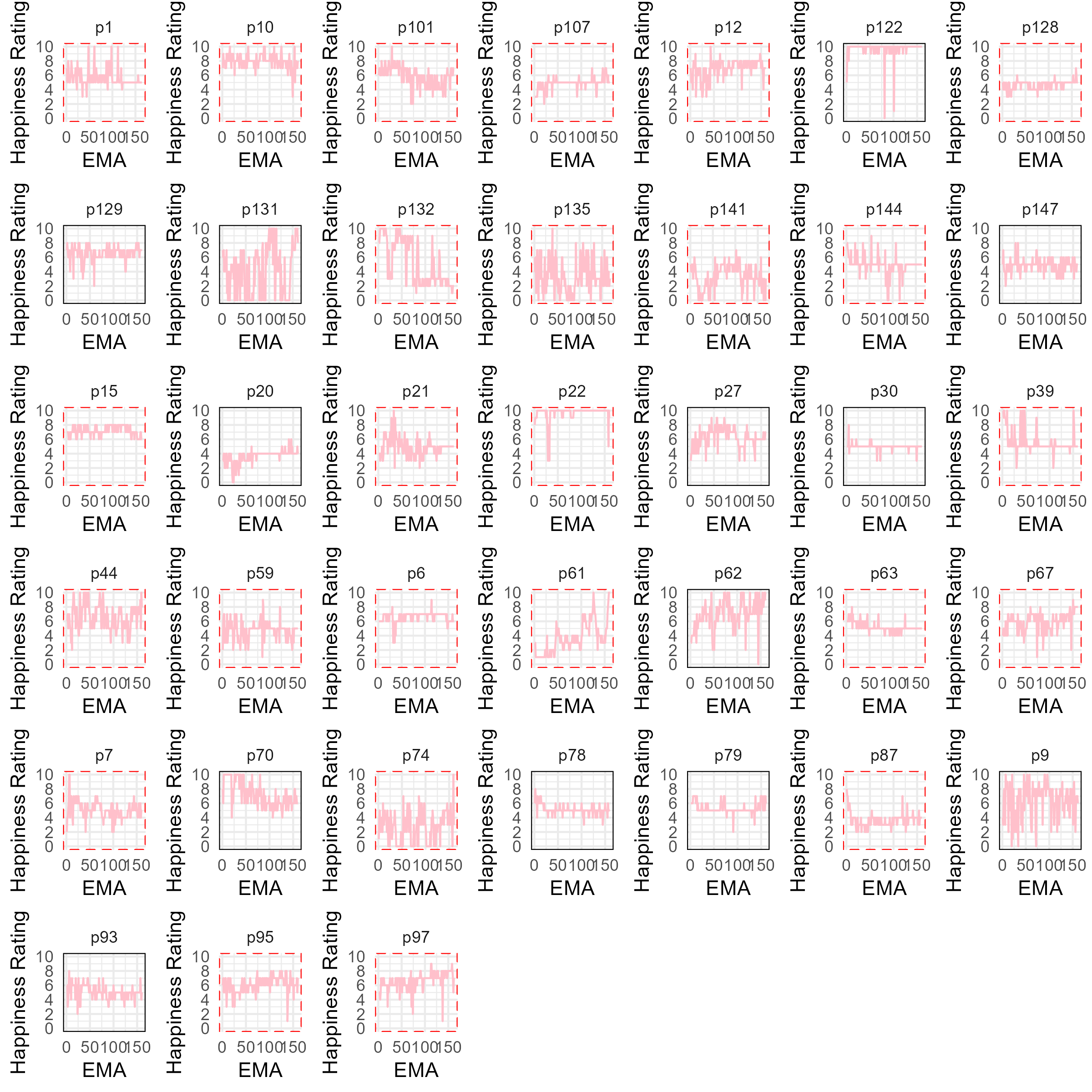


## Irritability


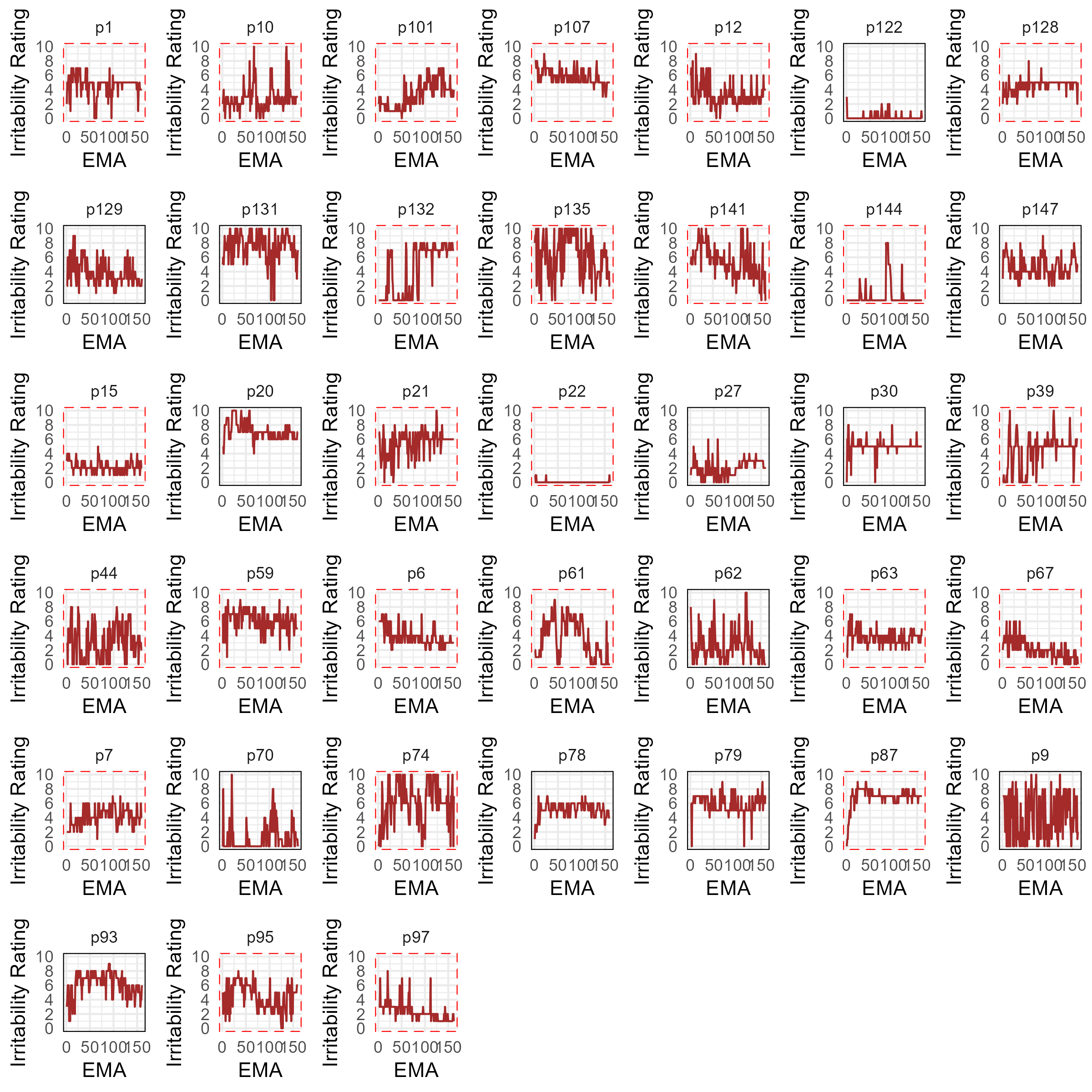


## Momentary motivation

##
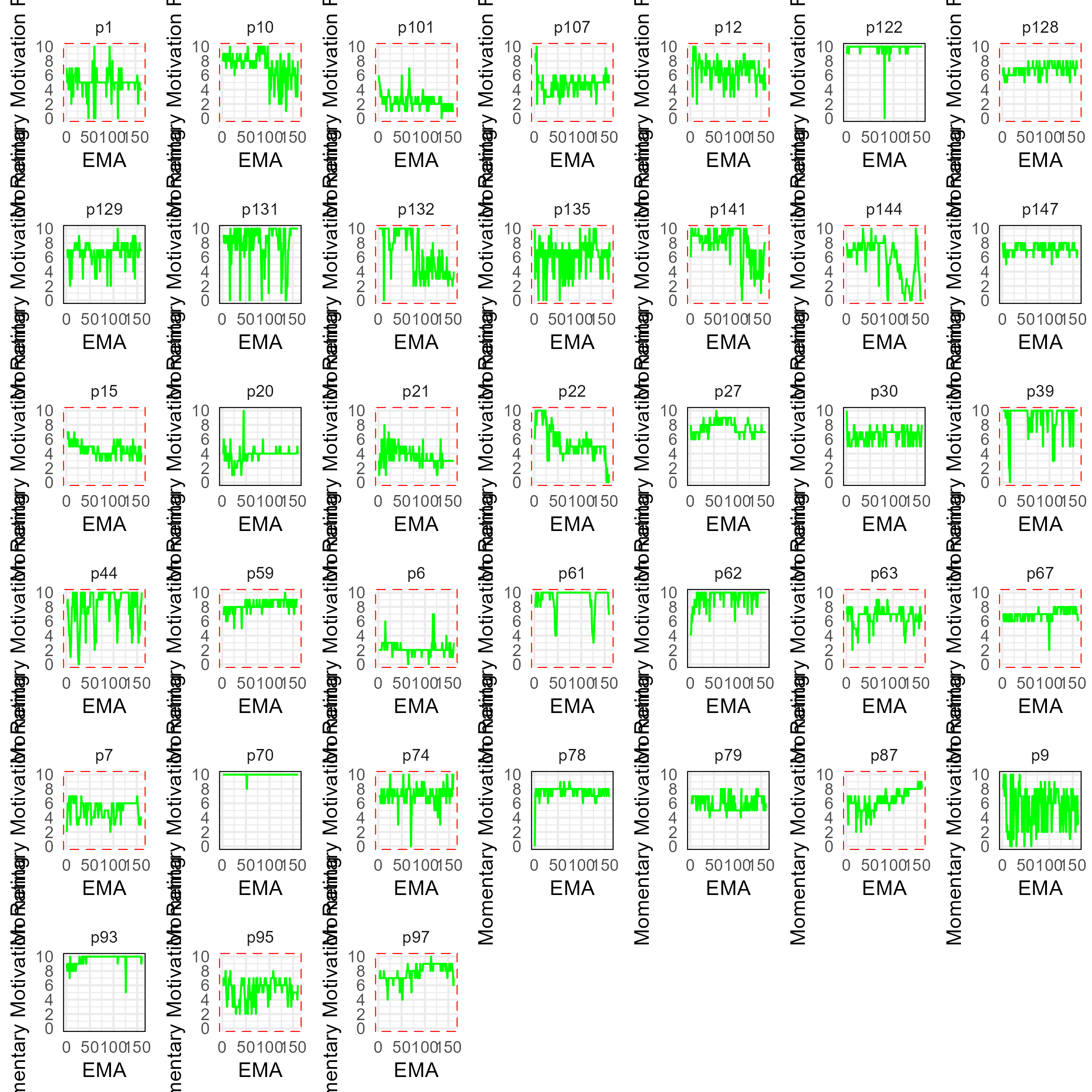


## Pain


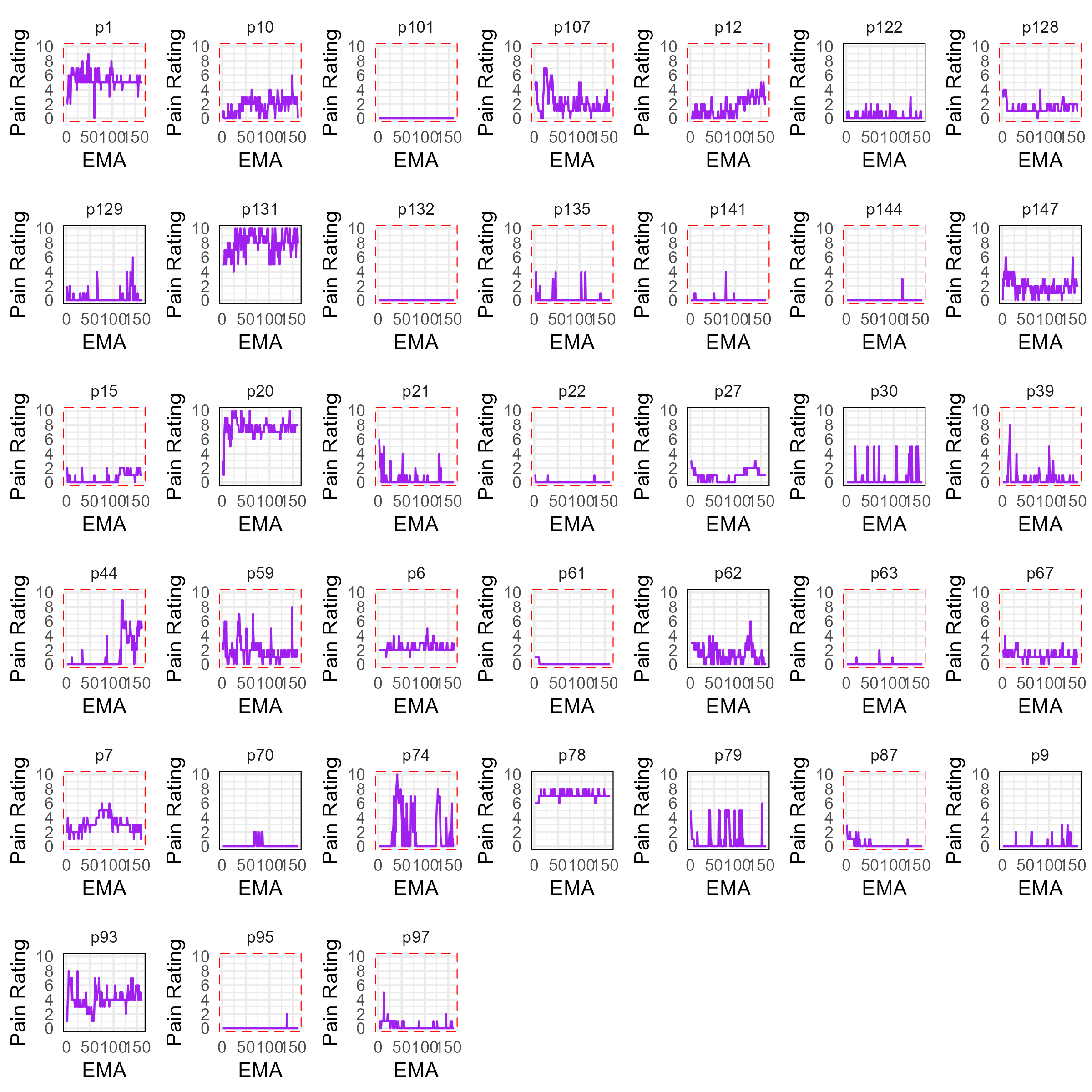


## Sadness


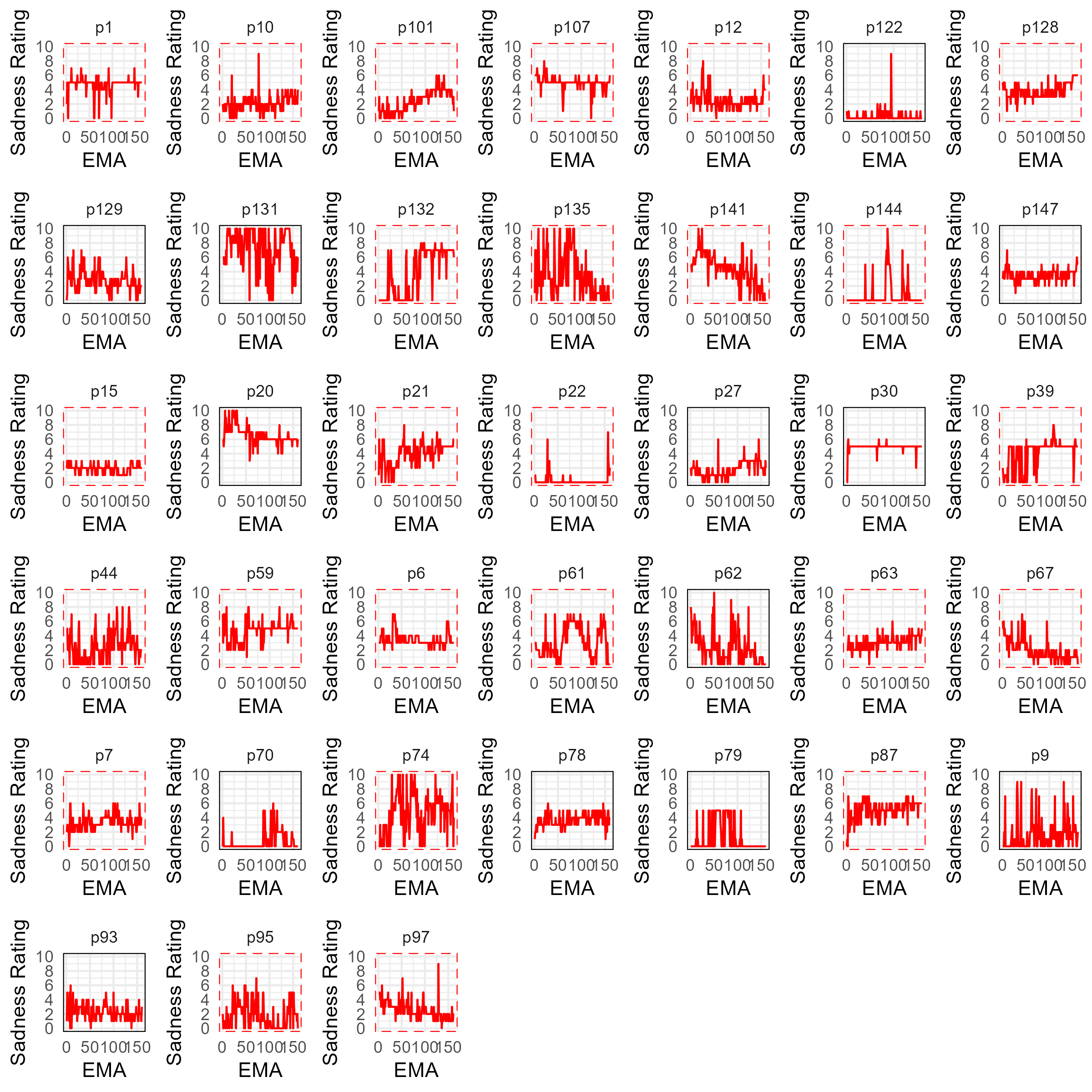


## Stress


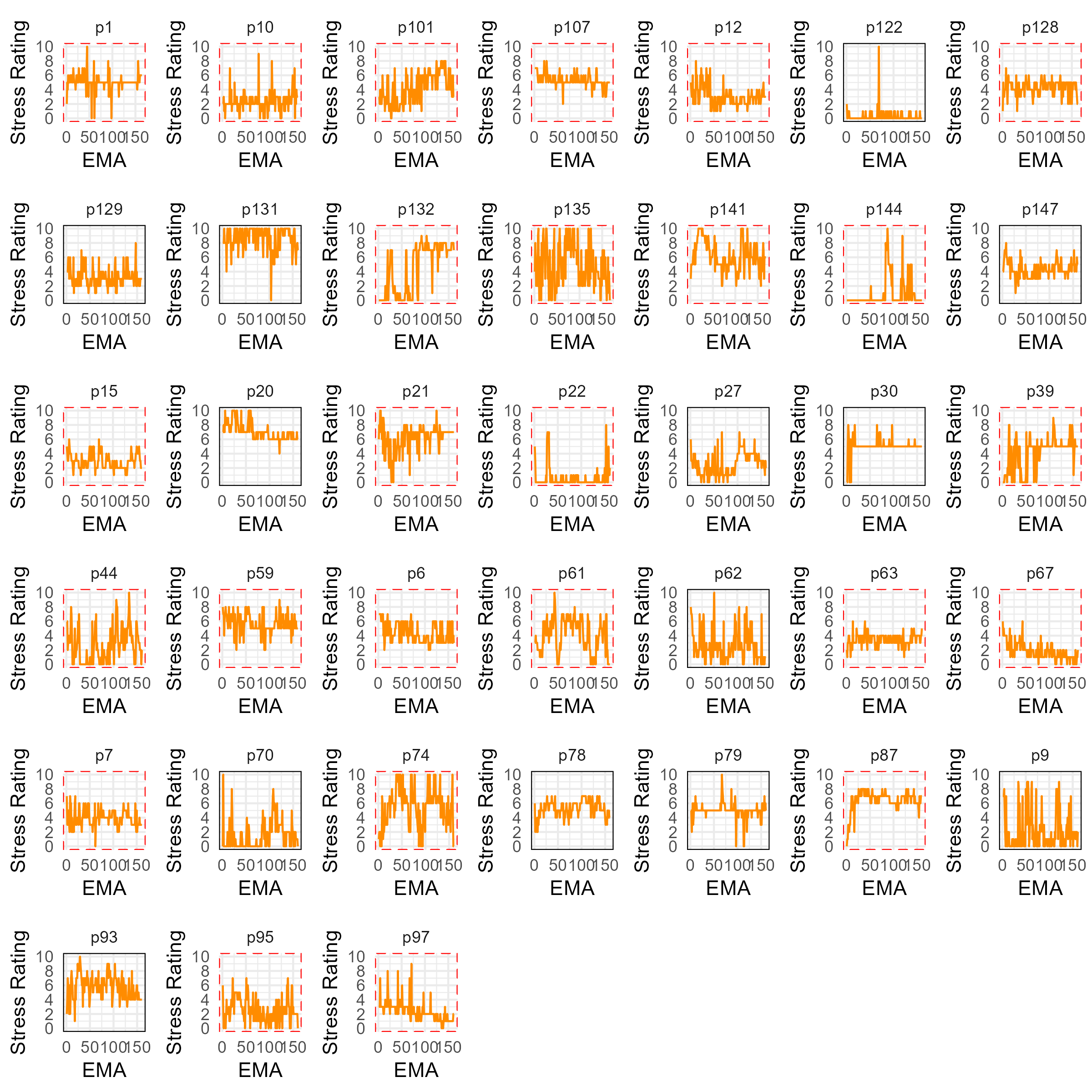


# Context variables

## Activities


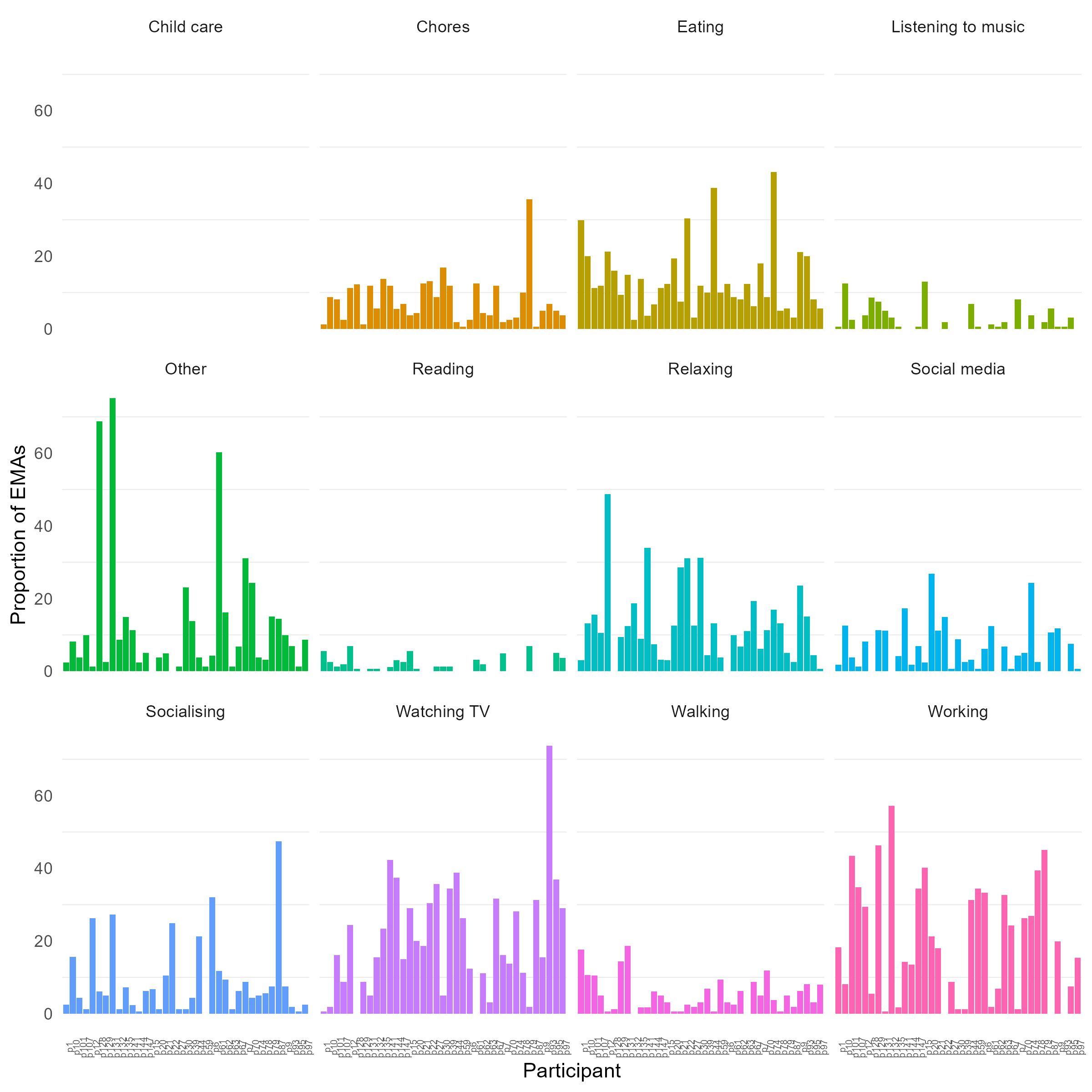


## Physical context


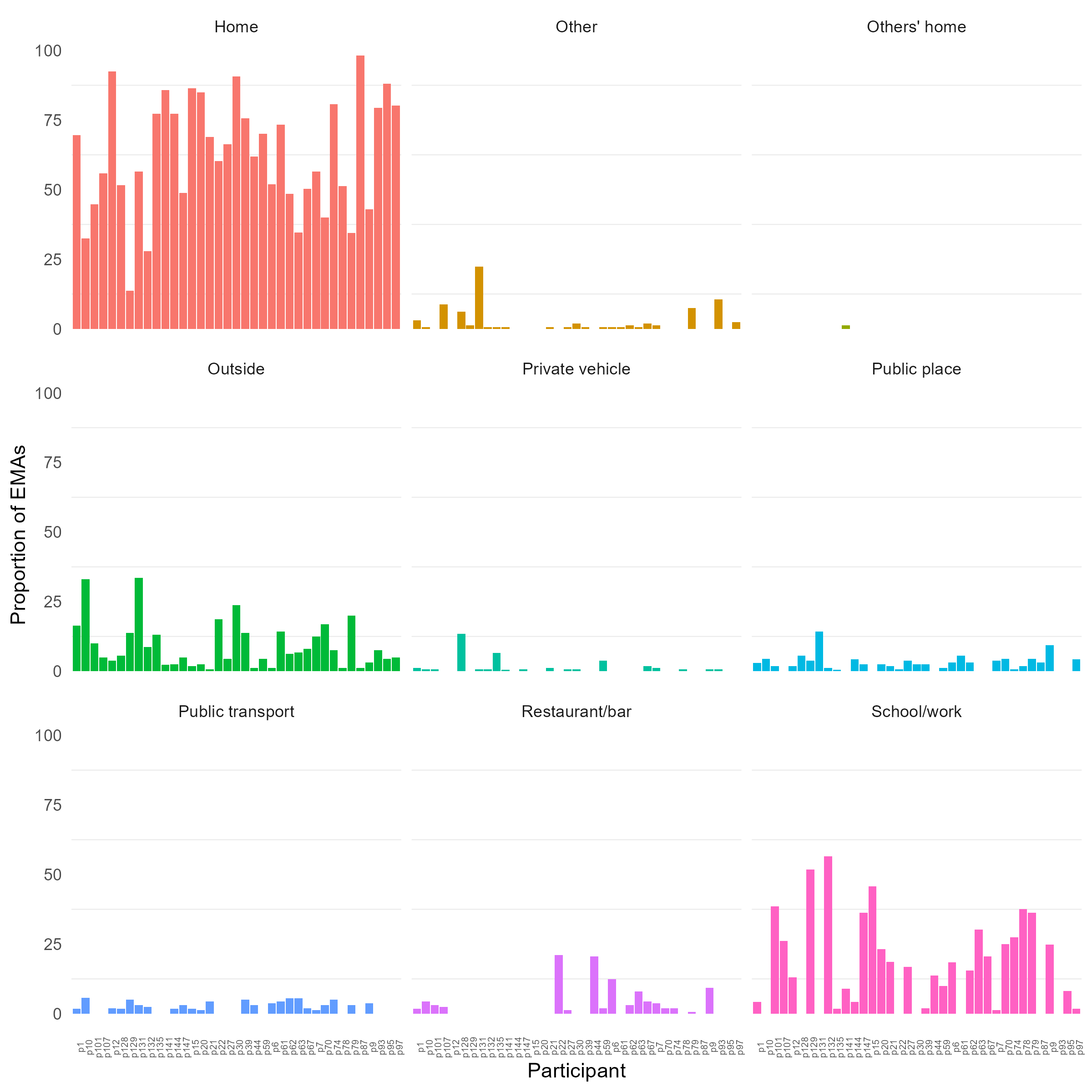


## Social context
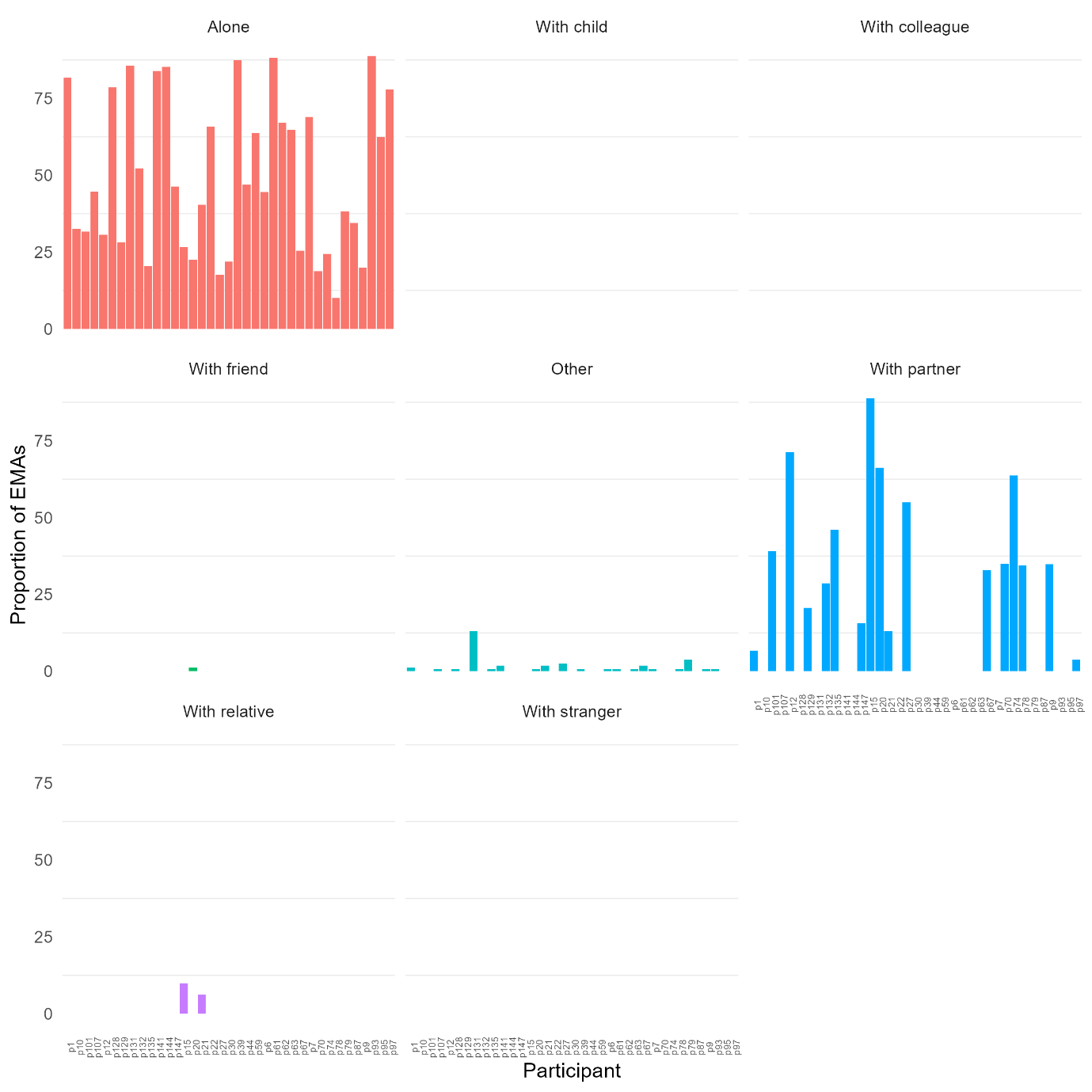

Supplement: S12 Appendix — (DOCX) [file pone.0349028.s012.docx]
